# The autopsy is not dead: ongoing relevance of the autopsy

**DOI:** 10.4322/acr.2023.425

**Published:** 2023-05-11

**Authors:** Alexia Vignau, Clara Milikowski

**Affiliations:** 1 University of Miami Miller School of Medicine, Miami, FL, United States of America; 2 University of Miami Miller School of Medicine, Faculty Clara Milikowski, Department of Pathology, Miami, FL, United States of America

**Keywords:** Clear cell carcinoma of ovary, hypercoagulability, B cell lymphoma, Guillain-Barré syndrome

## Abstract

**Background:**

Autopsy requests have been trending downward for a variety of factors. There are differences between pre- and postmortem diagnoses. Autopsies remain a tool for education, public health research, quality control, and closure for families.

**Objective:**

We report two cases that illustrate the utility of autopsy for uncovering contributing factors in the death of these patients and highlight their ongoing importance.

**Design:**

Clinical and autopsy investigation of two individuals and illustration of the importance of autopsy findings which, had they been diagnosed premortem, could have changed the outcome. Cases were evaluated using the Goldman criteria for discrepancies between premortem clinical diagnoses and postmortem autopsy findings.

**Results:**

In the first case, the patient had been previously admitted due to a non-ST elevation myocardial infarction months before the fatal event. The autopsy showed an undiagnosed clear cell carcinoma of the ovary. She expired due to a massive myocardial infarction secondary to neoplasm induced hypercoagulable state. The degree of pre-mortem/postmortem diagnostic discrepancy makes this a Goldman Class I error.

In the second case, the patient presented to the emergency department with symptoms of Guillain-Barré Syndrome (GBS), for which he was treated. Abdominal masses were discovered; however, the patient decompensated before workup was completed. A high-grade B-cell lymphoma was confirmed but would not have altered the outcome, making this a Goldman class II error.

**Conclusions:**

The autopsy remains a relevant and necessary tool for physicians and society. It assists in the establishment of diagnoses, measurement of treatment quality, the providence of public health metrics, and closure to the survivors.

## INTRODUCTION

Since the 1970s in the United States, autopsy rates have declined from being performed on 40-60% of hospital fatalities to less than 5%.^1^ Some have attributed the drop-in autopsy rate to its labor intensity and expense, resulting in a relative devaluation and priority for funding.^2^ Yet, in the ever-evolving world of medicine, the role of the autopsy remains as informative and pertinent as ever.

Despite technological advances which continue to increase the diagnostic tool set available to physicians, autopsies continue to be relevant. Goldman et al.^3^ reported on an extensive examination of autopsies over 3 decades. They found that 10% of autopsies over these decades showed a major diagnosis that may have led to a change in treatment and prolonged survival.^3^ The Goldman Classification (Table 1) categorizes cases into major discrepancies, which were related to the death, and minor discrepancies, which were incidental, in pre and postmortem findings, and whether the discovery of the findings premortem would have changed outcome. This classification system standardizes the evaluation of diagnostic accuracy and is important for ensuring the utmost quality of care.

**Table 1:** Goldman classification^3^

**Goldman Classification**	**Requirements**
**Class I**	Major discrepancy, where identification of diagnosis before death could have changed management and prolonged survival.
**Class II**	Major discrepancy in which identification of diagnosis would not have led to change in management/ survival.
**Class III**	Minor discrepancy where the disease was linked to terminal illness but ultimately did not directly cause death.
**Class IV**	Overlooked minor diagnoses that were important yet unrelated and could have eventually affected prognosis/outcome or a diagnosis that contributed to the passing of a terminally ill patient.

Beyond just for quality assurance and anatomical training for educational purposes, especially for pathology residents, the autopsy functions as a conduit for medical discovery and provides benefits to the family in the form of discovering heritable diseases and providing closure. Autopsies also offer benefits to society by detecting contagious diseases and environmental hazards. Discrepancies between premortem and postmortem diagnoses call for investigation and review to ensure that physicians are better able to hone their skillset and acumen to recognize symptoms that may fall outside of the typical patient presentation.

One recent domestic study reported a 26.1% discrepancy rate between ante and postmortem diagnoses, and another retrospective study in Brussels reported a 23.1% discrepancy.^4,5^ These studies support the findings of the retrospective study of the ongoing relevance of autopsies performed at Jackson Memorial Hospital in 2016, which found that 19.5% of the autopsies included in the study displayed major discrepancies in the pre and postmortem diagnoses (Goldman classification I and II).^6^ Here, we present two cases at the same institution which qualify as Goldman classification I and II discrepancies. The first case includes a woman who suffered from thrombotic events, which upon autopsy, were found to be a complication of clear cell carcinoma (CCC) of the ovary. The second case was a patient who was diagnosed with Guillain Barré Syndrome (GBS), which was found on autopsy to be a complication of undiagnosed non-Hodgkin’s lymphoma.

## CASE REPORTS

### Case 1

A 50-year-old otherwise healthy female with a 20-pack year smoking history presented to the emergency department (ED) in late 2021 with complaints of chest pain, tingling left arm, and dysarthria. This was diagnosed as a Non-ST Elevation Myocardial Infarction (non-STEMI). Cardiac catheterization did not show any obstructive coronariopathy. In addition, there was a concomitant embolic stroke as a result of a left ventricular thrombus. Initial coagulation studies showed PT of 17.2 sec (14.8 N), which subsequently normalized, INR, and PTT were within normal limits. Additional testing was negative for antinuclear antibody, lupus anticoagulant, and showed normal values for proteins C and S, Factor V, Cardiolipin IgG, Beta 2 glycoprotein IgG, and IgM, and antithrombin was wild type. The patient was stabilized, placed on a regimen of anticoagulation therapy with warfarin, and discharged.

The patient returned to the ED, two months after the first event, due to chest pain, hypotension, bradycardia, diaphoresis, and altered mental status. The patient was started on heparin, aspirin, and ticagrelor after an EKG showed a STEMI in the posterior leads prompting emergent cardiac catheterization. During the procedure, the patient coded and was transferred to the Cardiovascular Intensive Care Unit, where she decompensated, developed multi-organ failure, and was declared brain dead post neurological exam.

#### Autopsy Findings

**Figure 1:**
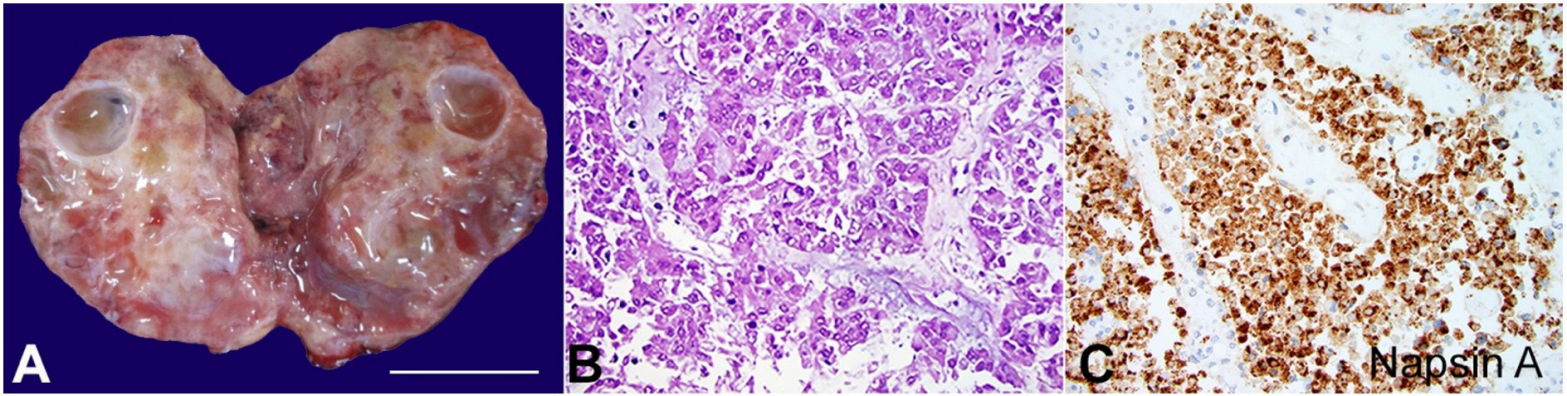
**A -** cross section of ovarian mass shows a solid gray-white gelatinous surface with few smooth walled cystic spaces and foci of hemorrhage (scale bar= 2 cm); **B -** the ovarian mass shows a pseudoglandular pattern with pleomorphic polygonal cells. Nuclei are vesicular and nuclear borders are irregular (H&E 400x); **C -** the tumor is diffusely positive for Napsin A (400x).

The autopsy was significant for a five-centimeter ovarian mass, an extensive myocardial infarction, and pulmonary embolus to the left main pulmonary artery. Cross section of the adnexal mass showed the tumor to be predominantly solid white with few smooth cystic spaces and foci of hemorrhage (Figure 1A). Histologically, the tumor showed a pseudoglandular pattern. The cells were pleomorphic and polygonal (Figure 1B). By immunohistochemistry, the cells were positive for PAX8, Napsin A (Figure 1C), WT1, and focally for CK7 and negative for CK20. These findings are consistent with clear cell carcinoma of the ovary.

**Figure 2:**
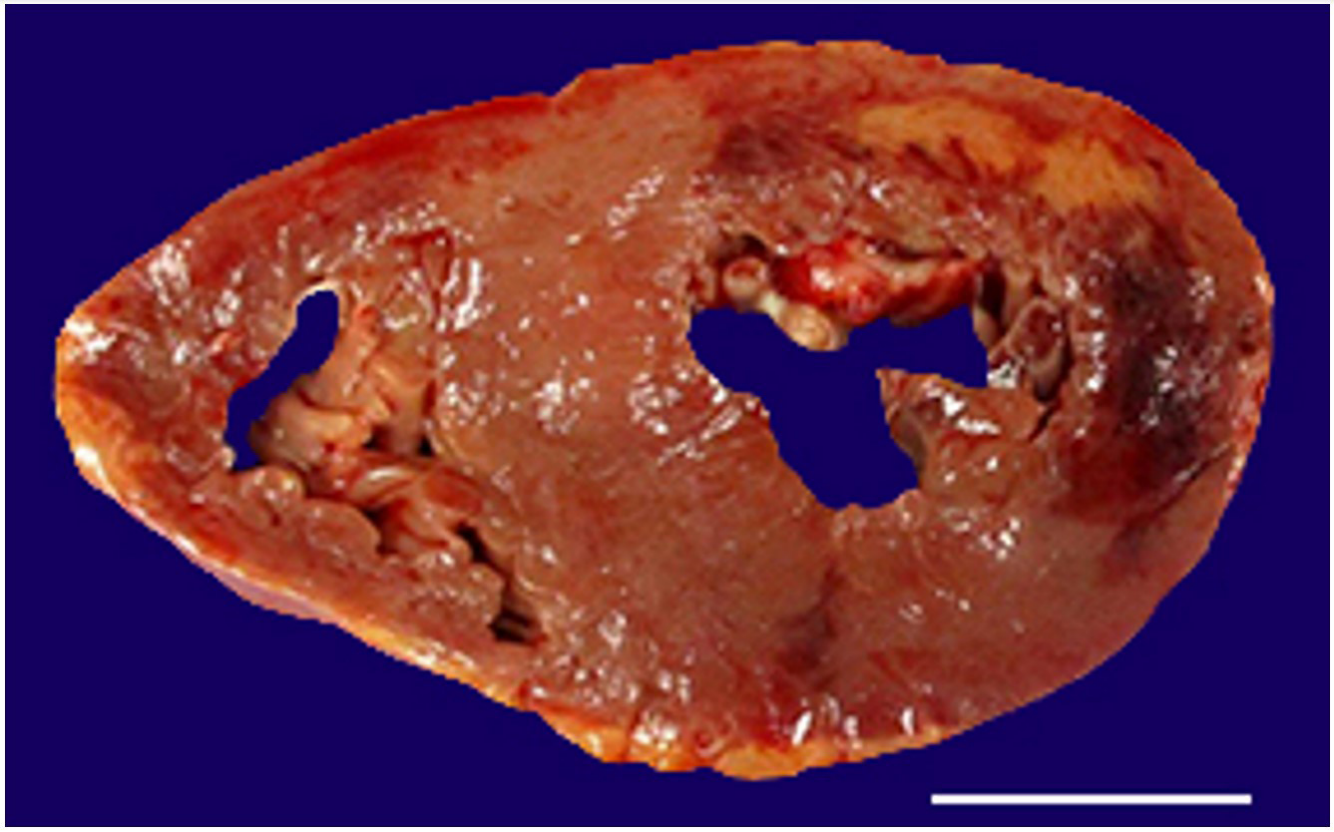
Cross section of heart shows a myocardial infarct involving the posterior and free walls of the left ventricle. The affected area is variegated ranging from bright red to yellow indicating various ages of the infarct (scale bar= 3.0 cm).

Cross section of the heart showed a large red to yellow area involving the free and posterior wall of the left ventricle (Figure 2), which extended from the apex to the base. The coronary arteries were thin, pliable, and patent. Histologically, the changes ranged from weeks to hyperacute.

The abdominal aorta showed few fatty streaks. The circle of Willis was complete, and the arteries were thin, pliable, and patent. Histologically, the brain showed diffuse cerebral edema and other findings consistent with reperfusion injury and “respirator” brain.

The left lung was involved by an organizing thrombus occluding the main pulmonary artery supplying the lower lobe with associated infarction. The clinicians requested the autopsy.

### Case 2

A 66-year-old male with a past medical history notable for several years of a cystic brain lesion presented to an outside hospital with complaints of bilateral forearm, left knee and ankle pain, right index finger numbness, right eye ptosis, overall weakness, and diplopia. He had received a COVID-19 booster shot one month prior. He was empirically treated for suspected GBS with intravenous immune globulin (IVIG) for five days and 8 days with acyclovir and showed some improvement in his symptoms. After completion of the IVIG, a spinal tap was performed, and cerebrospinal fluid analysis showed an elevated white blood cell count of 61/µL with 87% lymphocytes, elevated protein of 93 mg/dL and LDH of 34 U/L which suggested an infectious polyradiculitis versus a neoplastic polyradiculitis. Bacterial and viral panels by PCR were all negative. A cytologic evaluation was not performed. A hematology consultation was ordered due to anemia with a hemoglobin of 7 gm/dL, white blood cell count of 18 K/µL with 13.52 K/µL neutrophils. Seven days after finishing IVIG treatment, he developed signs of ileus. An abdominal x-ray confirmed paralytic ileus and a CT scan of the abdomen showed extensive mesenteric lymphadenopathy, most likely due to malignancy. There was no mention of groin lymph node enlargement. The following day he developed acute respiratory distress. CT scan of the chest showed a pulmonary embolism to the right lower lobe and there was no mention of axillary adenopathy. The patient was started on enoxaparin twice daily and he was intubated. A tissue biopsy of the mesenteric mass and a PET scan were ordered for diagnosis and staging; however, the patient was too ill to have the testing performed. Blood cultures came back positive for *Klebsiella pneumoniae*, and he was started on antibiotics. Eleven days post-intubation, hypotension and hypoxia worsened, vasopressor requirement increased, and he expired. The family requested the autopsy to clarify the disease and determine if there was a heritable component.

#### Autopsy Findings

**Figure 3:**
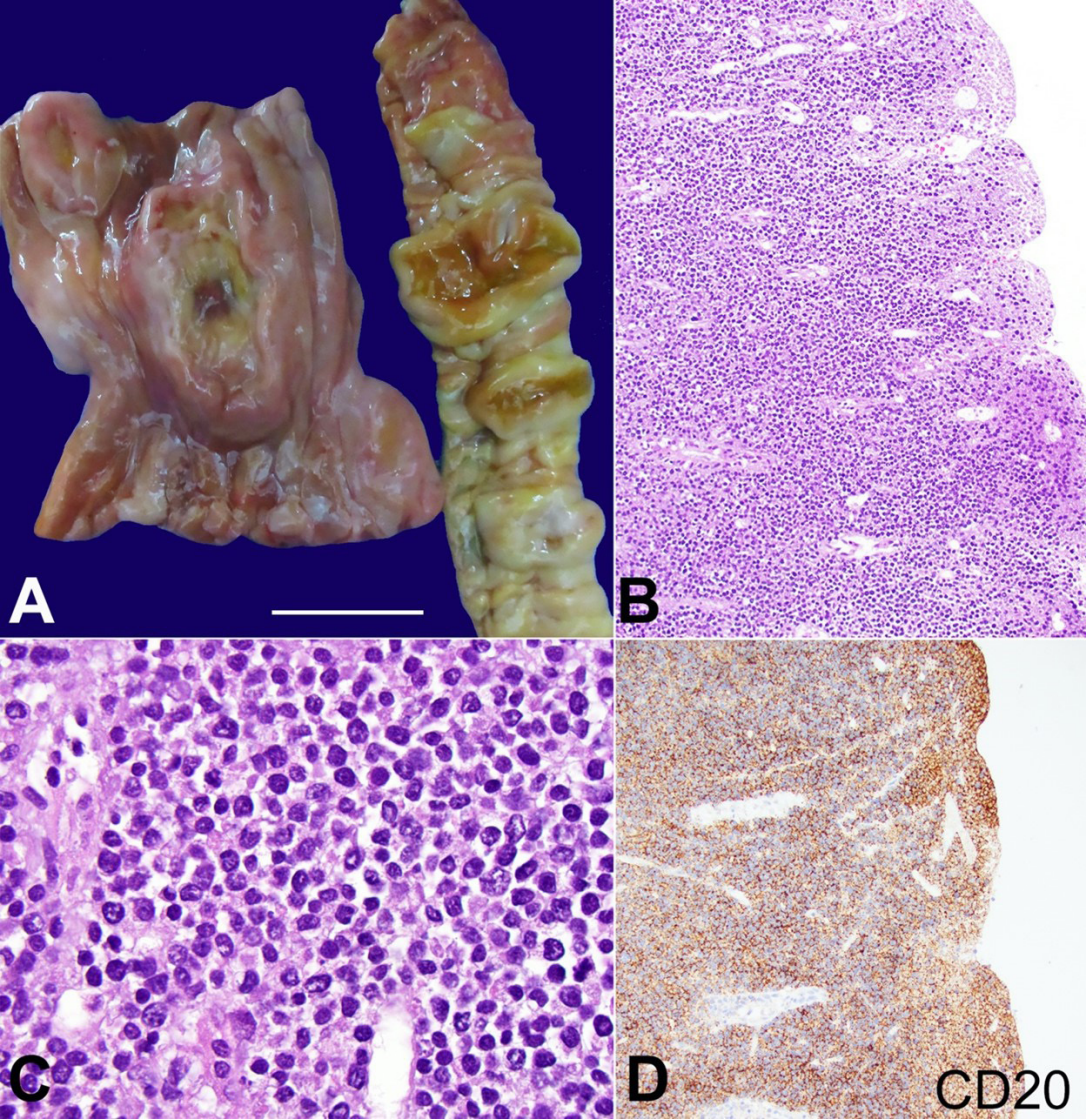
**A -** gross view of the stomach and small intestine involved by polypoid and ulcerated tumors; **B -** the wall of the small intestine is diffusely involved by lymphoma (H&E 200X); **C -** high power in better preserved area shows the lymphoma cells to be pleomorphic, intermediate to large size. (H&E 1000X); **D -** the tumor is diffusely positive for CD20 (200x).

At the abdominal cavity overture, there were many enlarged lymph nodes and tumor masses involving and infiltrating the mesentery, stomach, small intestine, left adrenal, and adipose tissue surrounding the cardiac circumflex artery. The lymph nodes and tumors on cut section were gray-white with a rubbery consistency. The stomach and small intestinal wall were thickened and essentially replaced by a white-tan tumor. The mucosa showed flat, polypoid nodular lesions with ulceration (Figure 3A). The enlarged lymph nodes in the root of the mesentery measured up to 3.4 cm with central necrosis. Gross examination of the brain was unremarkable.

Histologic sections showed small intestinal mucosa with overlying ulceration and diffuse involvement by neoplastic cells (Figure 3B). The cytology in better preserved areas, shows these cells to be intermediate-to-large in size (Figure 3C). By immunohistochemistry, these cells were positive for CD20 (Figure 3D), CD10, and PAX5 while negative for CD3, CD4, CD5, and CD138. The Ki-67 nuclear proliferation index > 75%. The overall findings are those of involvement by an aggressive B-cell lymphoma. Histologic sections of the brain showed an atypical lymphocytic infiltrate predominantly in the perivascular and subarachnoid spaces, periventricular white matter and basal ganglia. These findings are consistent with involvement by lymphoma.

## DISCUSSION

The autopsy rate has been declining since the 1970s in the U.S. and worldwide. This report is of two autopsy cases in which a premortem diagnosis would have changed treatment and eventually have changed the outcomes, or at least could have set physicians on the correct reasoning to treat or understand their patients' illness. Despite current sophisticated clinical investigations, autopsies remain an important adjunct in medicine. The presenting findings in these cases were not the usual presentations. In the first case, a thorough investigation into the causes of coagulopathy was not sought, and in the second case, the patient became too ill to have further investigation.

Physicians should be aware of and be able to recognize rarer presentations of disease processes. This level of discrimination can be the difference between life and death. Both cases presented, particularly the second one with GBS related to advanced B-cell lymphoma, seem to be more nuanced diagnoses in response to the neoplasm. However, earlier identification and treatment could have made a difference in survival.

In the case of the 50-year-old female with hypercoagulability secondary to her clear cell carcinoma of the ovary, earlier diagnosis and treatment may have altered the outcome. Understanding the underlying etiology of her symptoms and subsequent treatment of her cancer may have improved her prognosis, making this a Goldman Class I error. Venous thromboembolism is the second most common cause of death in cancer after cancer progression and should therefore be considered by oncologists treating cancer.^7^ The mechanism of Cancer-Associated Thrombosis is distinct from non-malignancy-associated thrombosis. The cancer tissue causes inflammation and coagulopathy due to the cascade that follows the activation of genes associated with cancer proliferation and metastasis.^8^ In fact, clear cell carcinoma is particularly notorious for the high incidence of thromboembolism due to tissue factor and interleukin-6 expression, possibly due to amplification of *MET* and *KRAS* genes.^9^ Seeing as the patient initially presented with an nonSTEMI and embolic stroke two months prior to her second hospital admission, empirical treatment of her primary diagnosis may have altered treatment strategies.

In the second case, the patient presented with what clinically appeared to be GBS. Autopsy revealed leptomeningeal involvement by his B cell lymphoma which was the cause of his symptoms. Despite not fully understanding the underlying diagnosis and etiology of this individual’s Guillain-Barré like symptoms, due to the rapid decompensation of the patient and his critical condition, the nuances of his diagnosis likely would not have made a difference in the outcome. Thus, the second case represents a Goldman class II error. GBS is the onset of ascending motor weakness due to neuronal demyelination in the peripheral nervous system.^10-12^CSF findings in GBS, first described by Guillain, Barré and Strohl in 1916 are distinctive (albuminocytologic dissociation) and characterized by an elevated CSF protein level (100-1000 mg/dL) with few lymphocytes, 10-50 at most.^10-12^ IVIG therapy may alter CSF findings by increasing protein and cell counts.^12^ A sustained CSF pleocytosis suggests an alternative diagnosis or a concurrent diagnosis such as unrecognized HIV infection, leukemia or lymphoma with infiltration of nerves.^12^ Non-Hodgkin lymphoma is rarely complicated by paraneoplastic syndromes, such as GBS.^11,13^ Leptomeningeal metastases occur in 3-8% of all cancer patients with solid tumors being most common but also occurring in hematolymphoid malignancies.^14^ Patients present with variable signs and symptoms which include pain and segmental weakness, loss of deep tendon reflexes, sensory loss, headache, gait abnormalities, mental changes, nausea, and vomiting.^14^ Signs of parenchymal involvement include aphasia, hemiparesis, and visual field defects.^14^ Many of these symptoms were manifest in the patient presented here. In the case presented, despite having an elevated white blood cell count, further diagnostic tests clarifying this diagnosis were not obtained due to rapid patient decompensation. Still, recognizing that GBS like symptoms could be a complication of lymphoma, can influence hospital teams’ running differential diagnosis and alter the prioritization of certain diagnostic exams for future patients.

## CONCLUSION

Ultimately, autopsy remains a necessary tool to sharpen and diversify physicians’ differentials and act as a quality measure for diagnostic accuracy. Earlier consideration of these rarer presentations in both cases could have determined a different course of action. In addition, though these presentations may be rare, it is difficult to truly know their prevalence with certainty without considering that cases may go undiagnosed. Increasing awareness of diverse illness presentations ensures that physicians treat people (personalized medicine) and not just patterns.

Additionally, the clinician or pathologist may have a postmortem autopsy conference with the family and play a role in the grieving process by providing clarification of the underlying cause of death, and alleviate guilt in insuring that the proper care was rendered to the patient, especially in cases of sudden and unexpected death.^15^ For instance, autopsy findings could provide closure and possibly alert family members of hereditary conditions and predispositions. In terms of public health, clarification of the cause of death could also help track infectious disease spread and mortality rates or the presence of environmental hazards. Thus, autopsy remains an important way to establish diagnoses, measure treatment quality, provide public health metrics for the community and provide closure to those surviving the patient.

## References

[1] Hamza A (2017). Declining rate of autopsies: implications for anatomic pathology residents. Autops Case Rep.

[2] Dehner LP (2010). The medical autopsy: past, present, and dubious future. Mo Med.

[3] Goldman L, Sayson R, Robbins S, Cohn LH, Bettmann M, Weisberg M (1983). The value of the autopsy in three medical eras. N Engl J Med.

[4] Rusu S, Lavis P, Salgado VD (2021). Comparison of antemortem clinical diagnosis and post-mortem findings in intensive care unit patients. Virchows Arch.

[5] Kurz SD, Sido V, Herbst H (2021). Discrepancies between clinical diagnosis and hospital autopsy: a comparative retrospective analysis of 1,112 cases. PLoS One.

[6] Marshall HS, Milikowski C (2017). Comparison of clinical diagnoses and autopsy findings: six-year retrospective study. Arch Pathol Lab Med.

[7] Fernandes CJ, Morinaga LTK, Alves JLJ (2019). Cancer-associated thrombosis: the when, how and why. Eur Respir Rev.

[8] Mukai M, Oka T (2018). Mechanism and management of cancer-associated thrombosis. J Cardiol.

[9] Tamura R, Yoshihara K, Enomoto T (2022). Therapeutic strategies focused on cancer-associated hypercoagulation for ovarian clear cell carcinoma. Cancers.

[10] Shahrizaila N, Lehmann HC, Kuwabara S (2021). Guillain-Barré syndrome. Lancet.

[11] Seffo F, Daw HA (2010). Non-Hodgkin lymphoma and Guillain-Barré syndrome: a rare association. Clin Adv Hematol Oncol.

[12] Sheikh KA (2020). Guillain-Barré syndrome. Continuum.

[13] Jiang QL, Pytel P, Rowin J (2010). Disseminated intravascular large-cell lymphoma with initial presentation mimicking Guillain-Barré syndrome. Muscle Nerve.

[14] DeAngelis LM (1998). Current diagnosis and treatment of leptomeningeal metastasis. J Neurooncol.

[15] McPhee SJ, Bottles K, Lo B (1986). To redeem them from death. Reactions of family members to autopsy. Am J Med.

